# Green tea polyphenol tailors cell adhesivity of RGD displaying surfaces: multicomponent models monitored optically

**DOI:** 10.1038/srep42220

**Published:** 2017-02-10

**Authors:** Beatrix Peter, Eniko Farkas, Eniko Forgacs, Andras Saftics, Boglarka Kovacs, Sandor Kurunczi, Inna Szekacs, Antal Csampai, Szilvia Bosze, Robert Horvath

**Affiliations:** 1 Doctoral School of Molecular and Nanotechnologies, Faculty of Information Technology, University of Pannonia, H-8200 Egyetem u. 10, Veszprém, Hungary; 2 Nanobiosensorics Group, Hungarian Academy of Sciences, Research Centre for Natural Sciences, Institute for Technical Physics and Materials Science, Konkoly-Thege u, 29-33, H-1120 Budapest, Hungary; 3 Chemical Engineering and Material Science Doctoral School, University of Pannonia, H-8200 Egyetem u, 10, Veszprém, Hungary; 4 George Olah Doctoral School, Faculty of Chemical Technology and Biotechnology, Budapest University of Technology and Economics, Műegyetem rkp. 3, Budapest 1111, Hungary; 5 Institute of Chemistry, Eötvös Loránd University, Budapest 112, POB 32, H-1518, Hungary; 6 MTA-ELTE Research Group of Peptide Chemistry, Hungarian Academy of Sciences, Eötvös Loránd University, Budapest 112, POB 32, H-1518, Hungary

## Abstract

The interaction of the anti-adhesive coating, poly(L-lysine)-*graft*-poly(ethylene glycol) (PLL-*g*-PEG) and its Arg-Gly-Asp (RGD) functionalized form, PLL-*g*-PEG-RGD, with the green tea polyphenol, epigallocatechin-gallate (EGCg) was *in situ* monitored. After, the kinetics of cellular adhesion on the EGCg exposed coatings were recorded in real-time. The employed plate-based waveguide biosensor is applicable to monitor small molecule binding and sensitive to sub-nanometer scale changes in cell membrane position and cell mass distribution; while detecting the signals of thousands of adhering cells. The combination of this remarkable sensitivity and throughput opens up new avenues in testing complicated models of cell-surface interactions. The systematic studies revealed that, despite the reported excellent antifouling properties of the coatings, EGCg strongly interacted with them, and affected their cell adhesivity in a concentration dependent manner. Moreover, the differences between the effects of the fresh and oxidized EGCg solutions were first demonstrated. Using a semiempirical quantumchemical method we showed that EGCg binds to the PEG chains of PLL-*g-*PEG-RGD and effectively blocks the RGD sites by hydrogen bonds. The calculations supported the experimental finding that the binding is stronger for the oxidative products. Our work lead to a new model of polyphenol action on cell adhesion ligand accessibility and matrix rigidity.

Detection of cellular adhesion is of outstanding diagnostic and basic research utility. On the one hand, changes in cell adhesivity can be a sign for various diseases; e.g. the variety of integrins, a major family of cell adhesion receptors that bind to the extracellular matrix (ECM), changes during tumor transformation^1^. On the other hand, measurement of the effect of bioactive substances on the adhesion of cancer cells can be an effective tool in the design of antineoplastic pharmaceuticals. By controlling interactions between a cell and its ECM, cell behavior and function can be influenced^2^. Under *in vivo* conditions, the cell adhesion involves several components, these are interacting in a complicated and tightly controlled manner, still under intense research. These components are the proteins and carbohydrates of the extracellular matrix, the cell adhesion receptors and other soluble factors (ions, small molecules) regulating the interactions. In contrast, due to experimental difficulties, most experimental models resulting in quantitative data about the cellular adhesion can be considered as a strong simplification of the *in vivo* situation.

A wide range of experimental methods are available to measure cell adhesion and cell–surface interactions^3^,^4^,^5^,^6^,^7^,^8^. However, most of them have serious disadvantages when a multicomponent model of cell adhesion has to be quantitatively investigated in a reasonable time frame. For example, labeling techniques use fluorescent markers that may affect normal cell behavior and the imaging time is often limited by the bleaching of the marker. Furthermore, dyes may interact with the sample material itself. Some techniques usually involve complicated and time-consuming steps and are not available in high-throughput format. Consequently, it is difficult to do large number of parallel measurements simultaneously, and sometimes it can easily take months to execute all of the experiments required^9^,^10^,^11^.

Label-free biosensors, not requiring the applications of fluorescent dyes, have the potential to become a common tool for measuring cell adhesion, spreading, proliferation, cellular differentiation, migration, receptor–ligand binding, signal transduction analysis and cytotoxicity. These techniques are especially promising when the kinetics of interactions have to be investigated. Sensitivity and detection capacity used to be considered as obstacles of the widespread use of label-free detection^12^, but recent developments have by far overcome these limitations. While quartz crystal microbalance (QCM)^4^,^6^,^13^, cellular dielectric spectroscopy (CDS)^14^,^15^, optical waveguide lightmode spectroscopy (OWLS)^16^, surface plasmon resonance (SPR)^7^ usually employ one or a low number of sensing units, novel biosensors have high-throughput capability to practically parallel measurements of hundreds of samples in a microplate format. At present, they easily meet the required sensitivity of being able to detect the binding of ligands of molecular mass as low as 100–200 Da, below 5 pg/mm^2^ surface mass density; and their current throughput allows up to 460,000 data points/hour. These include electric cell–substrate impedance sensing (ECIS)^5^,^4^,^17^, photonic crystal based sensors^18^,^19^, and resonance waveguide grating (RWG)^8^,^11^,^20^. Moreover, it has been proven that optical waveguide based sensors are capable of investigating not just biological samples, but nanoparticles and self-assembled nanostructured coatings as well^21^,^22^.

PLL-*g*-PEG is a polycationic copolymer with PEG chains covalently grafted onto a positively charged (at pH 7) PLL backbone. The copolymer spontaneously adsorbs from aqueous solution via electrostatic interactions onto negatively charged surfaces such as Nb_2_O_5_, SiO_2_, TCPS, TiO_2_; but recent studies have shown that PLL-*g*-PEG can also adsorb onto nonpolar, hydrophobic polydimethylsiloxane (PDMS) surfaces from aqueous solutions^23^,^24^. The PEG side chains stretch into the bulk aqueous solution to generate a brush-like conformation because of its high affinity for water. Previous studies have shown that a PLL-*g*-PEG layer on various metal oxide surfaces displays excellent resistance to non-specific adsorption of proteins and cell adhesion, and provides a lubricious surface in an aqueous environment^24^,^25^. The copolymer and its cell adhesive, functionalized counterpart, PLL-*g*-PEG-RGD can be mixed to vary the cell adhesion ligand density at the nanoscale, and to induce cell adhesion and spreading on the coated surfaces. Here, we use the mixture of PLL-*g*-PEG and PLL-*g*-PEG-RGD to control the spreading abilities of the monitored cells^26^,^27^. Note, PLL-*g*-PEG is often considered as a gold standard of antifouling surfaces, but the interaction of these coatings with polyphenols has never been investigated.

Tea catechins, especially EGCg, have been shown to have various health benefits, for example anti-metastasis, anticancer, anti-inflammatory and antioxidant properties, prevent cardiovascular disease^28^,^29^,^30^. EGCg is one of the most studied active substances, and many studies observed its effects on several cancer and normal cell lines, and on animal models. In the studied cell lines, this compound had significant impact on cell adhesion and movement, apoptosis, proliferation generally by altering gene expression^31^,^32^,^33^,^34^,^35^,^36^,^37^. Tea polyphenols are well known for their antioxidant activities, too^38^. Among them, EGCg is the most effective compound interacting with reactive oxygen species (ROS)^39^. EGCg and other catechins are unstable at high temperatures and under alkaline and neutral conditions, at pH above 7 EGCg oxidizes and dimerizes easily^38^,^40^. In aqueous solution, it changes from non-colored at around natural pH to yellow at higher pH region, and its absorption in the UV range became more pronounced^41^. This oxidation is an irreversible reaction, and Mizooku and co-workers revealed that the oxidation species was found to correspond to the molecular weight of M_w_ + 14 Da (where M_w_ is the molecular weight of EGCg), which has two hydrogen atoms removed and addition of one oxygen atom to the galloyl moiety in the B-ring of EGCg^41^. EGCg is known to be unstable under cell culture conditions, autooxidation and H_2_O_2_ generation are induced as well^41^,^42^,^43^,^44^, however, formation of H_2_O_2_ was observed in aqueous solutions, too^41^. The reaction is propagated by the reaction of superoxide with EGCg, generating EGCg dimers and H_2_O_2_^41^. The dimers can be further transformed to other compounds, supposedly polymers^43^. The major oxidative products are theasinensin A (C44H34O22, molecular weight 914 Da), and another dimer with a molecular weight of 884 Da^38^,^43^,^45^. Both are dimers of EGCg, which have been reported to be formed in mild alkaline fluids or after radical reaction with 1,1-diphenyl-2-picryl-hydrazyl radical, and show brown, yellow color^38^,^46^. Despite the massive number of studies, remarkably, its interaction with cell adhesive or anti-adhesive coatings was rarely investigated^23^,^25^. The direct influence of EGCg on the adhesive coatings seems to be neglected in the literature. This could be due to the experimental difficulties arising when working with EGCg. First, it has a rather small molecular weight of 458.40 Da, making it difficult to detect. Second, the molecule is unstable and is a subject of oxidation and polymerization.

In the present work, we highlight the remarkable potentials of high-throughput resonant waveguide gratings in studying multicomponent model systems of cell–surface interactions. The interaction of the anti-adhesive, antifouling coating, PLL-*g*-PEG and its RGD (Arg-Gly-Asp) functionalized form, PLL-*g*-PEG-RGD, with the green tea polyphenol, EGCg was *in situ* monitored. Right after, cellular adhesion on the EGCg exposed coatings was recorded in real-time. The plate based sensor configuration allowed following the above processes with different surface coatings, EGCg states and concentrations in a single run, on the same biosensor plate. Despite the reported excellent antifouling properties of the above polymer coatings, EGCg strongly interacted with them, and affected their cell adhesivity in a concentration dependent manner. The differences between the effects of the freshly prepared and oxidized EGCg solution could be also first demonstrated. The measured interactions were significantly stronger for the oxidized EGCg solution, highlighting the importance of storage conditions of EGCg solutions, often overlooked in present literature. Using a semiempirical quantumchemical method we showed that EGCg binds to the PEG chains of PLL-*g*-PEG and PLL-*g*-PEG-RGD by hydrogen bonds. Moreover, the calculations illuminated the differences in binding affinity between the fresh and oxidized EGCg, well supporting the experimental findings.

## Materials and Methods

### Synthetic polymer solutions

The synthetic copolymers, poly(L-lysine)-*graft*-poly(ethylene glycol) (PLL-*g*-PEG, [PLL(20)-g(3.5)-PEG(2)]) (hereafter PP) and its RGD functionalized counterpart, PLL-*g*-PEG/PEG-GGGGYGRGDSP (PLL-*g*-PEG-RGD [PLL(20)-g(3.5)-PEG(2.3)/PEG(3.4)-RGD]) (hereafter PPR) were obtained as powders from SuSoS AG, Dübendorf, Switzerland. The materials were stored at -20 °C until use. Each powder was then dissolved in 10 mM 4-(2-hydroxyethyl)−1-piperazine ethanesulfonic acid (HEPES, from Sigma-Aldrich Chemie GmbH, Munich, Germany) at pH 7.4 to make stock solutions with a concentration of 1.0 mg/ml. Coating solution with RGD-motifs and PLL-*g*-PEG were prepared by mixing the two 1 mg/ml stock solutions (hereafter PP:PPR)^27^.

### Preparation of EGCg and oxidized EGCg solutions

The EGCg powder (from Sigma-Aldrich Chemie GmbH, Munich, Germany) was solved in 10 mM HEPES at pH 7.4, or in assay buffer (20 mM HEPES in HBSS, pH 7). The concentration of the dissolved EGCg solution was 0.05, 0.5, 5, 20, 40, 50 and 500 μg/ml. Oxidation process was conducted by simply exposing the prepared EGCg solutions to higher temperature (for example room temperature), and as a result, we got brown, oxidized liquids^38^,^40^,^41^. To get oxidized EGCg solutions in highly reproducible manner, the freshly prepared solutions underwent a heat treatment at 70 °C for 1.5 h.

### Cell culture and cell adhesion assay buffer

HeLa cells were routinely cultured in tissue culture polystyrene Petri dishes (Greiner Bio-One International GmbH, Kremsmünster, Austria) placed in a humidified incubator (37 °C, 5% CO_2_). The cells were maintained in Dulbecco’s modified Eagle’s medium (DMEM), supplemented with 10% fetal bovine serum (Biowest SAS, France), 4 mM L-glutamine (from Sigma-Aldrich Chemie GmbH, Munich, Germany), 0.25 μg∕ml amphotericin B (from Sigma-Aldrich Chemie GmbH, Munich, Germany), 100 U∕ml penicillin (from Sigma-Aldrich Chemie GmbH, Munich, Germany) and 100 μg∕ml streptomycin solution (from Sigma-Aldrich Chemie GmbH, Munich, Germany). On reaching 80% confluence, cells were detached every 3–5 days using 0.05% (w/v) trypsin (from Sigma-Aldrich Chemie GmbH, Munich, Germany), 0.02% (w/v) EDTA solution (from Sigma-Aldrich Chemie GmbH, Munich, Germany) and were not used beyond passage 20^47^. Cell adhesion assay buffer was prepared by adding 20 mM HEPES to Hank’s balanced salt solution (HBSS, from Sigma-Aldrich Chemie GmbH, Munich, Germany) and adjusted to pH 7.0 with 1 mM NaOH (hereafter HBSS-HEPES).

### OWLS for *in situ* monitoring the formation of polymer layers and subsequent EGCg adsorption

OWLS is a label-free technique that uses evanescent optical waves^48^. During the experiment, linearly polarized light is coupled into a planar optical waveguide sensor chip (type OW2400, Microvacuum Ltd., Hungary) through a coupling grating. The OWLS instrument (BIOS210, Microvacuum Ltd.) records the effective refractive indices (*N*) of the zeroth-order transverse electric (TE) and transverse magnetic (TM) polarized waveguide modes with a time resolution of ∼13 s. Upon adsorption onto the surface of the sensor, the effective refractive indices shift to higher values, allowing monitoring of the *in situ* kinetics of adsorption processes. OWLS has been mostly used to characterize surface adsorption properties in protein–substrate or protein–nanoparticle film interactions^21^.

Before the measurements, the OWLS chip was immersed in chromosulfuric acid and potassium hydroxide to clean its surface. The plastic cuvette and the fluidic system were treated by oxigen plasma (SPI Supplies Plasma Prep II) to remove possible contamination remained from the previous experiment^9^,^21^,^49^.

During the OWLS measurement of the adsorption of the PP coating, first, the baseline was recorded with HEPES buffer by using a peristaltic pump generated flow (1 μl/sec) for approximately 40 min. After, PP was injected (100 μl) for 20 min to create the coating. The excess PP was removed with HEPES buffer pumped by using a peristaltic pump (1 μl/sec) for approximately 20 min. After, 100 μl EGCg solution (500 μg/ml, solved in HEPES buffer) was injected for 20 min. Then another washing period was performed with HEPES buffer by peristaltic pump (1 μl/sec) for approximately 20 min. In case of the PP:PPR coating, first, the baseline was recorded with continuous flow of HEPES buffer using peristaltic pump with the rate of 1 μl/sec for approximately 40 min. After that 100 μl of PP:PPR was injected onto the sensor surface to create the coating and washed out with HEPES buffer after 20 min incubation. Then the HEPES buffer was exchanged to HBSS-HEPES buffer and the signal was recorded for 20 min, until the stabilization of the baseline. After, 100 μl of EGCg solution (500 μg/ml, in HBSS-HEPES buffer) was injected to the cuvette and incubated for 20 min, then the excess was washed out by HBSS-HEPES.

### The Epic BT biosensor and related protocols for *in situ* monitoring the polymer adsorption, EGCg binding and cell adhesion

The Epic BT system (Corning Incorporated, NY, USA) employed in the present study is a next-generation resonant waveguide grating imager biosensor allowing high-throughput label-free detection at a solid–liquid interface. It works with 96- or 384-well biosensor microplates. In this work, a 384-well uncoated Epic microplate (5040, Corning) was used. The bottom of an Epic microplate serves as a planar optical waveguide, a thin, high refractive index, transparent dielectric layer (waveguide layer, made of the biocompatible material Nb_2_O_5_) on a thicker substratum. At the central position of each well, an optical grating is embedded into the waveguide layer to enable the incoupling of the illuminating light; thus separate biosensors are created. Incoupled light beams undergo total internal reflections at the inner surfaces of the waveguide layer, and gain a phase shift upon each reflection. The extent of the acquired phase shift is dependent on the refractive index (RI) of the medium being closest to the reflecting surface (because an exponentially decaying electromagnetic field, called an evanescent field, penetrates into an approximately 150 nm thick layer of the neighboring medium and probes the local RI). Light beams incoupled by the same grating interfere with each other, but only constructive interference results in waveguiding. This criterion is met only at a certain illuminating wavelength, called the resonant wavelength (λ). Any process accompanied by RI-variations in the approximately 150 nm thick layer over the biosensor surface (bulk RI change, molecular adsorption, cell spreading, or dynamic mass redistribution in the cells) alters the acquired phase shift when the beams undergo reflections at the waveguide layer–sample interface. This untunes the resonance, but waveguiding can resume at another illuminating wavelength (λ’≠λ). The primary signal output of the Epic BT system is the shift of the resonant wavelength (Δλ = λ’-λ) in each well. Δλ is proportional to the alteration in the effective refractive index *N* of the substrate-cell-medium system. At constant cell number and cell volume, *N* is related in a simple way to the degree of spreading. It should be emphasized that non adhered round shaped cells do not contribute to the signal, since the amount of material entering into the evanescent fields is negligible in this case^48^. In practice, all wells of an Epic microplate are simultaneously interrogated at every 3 seconds by sweeping the illuminating wavelength through a range of 15000 pm with 0.25 pm precision^27^. Of note, this resolution corresponds to a minimum detectable surface bound amount of 0.078 ng/cm^2^, the system is applicable for fragment based screening^50^.

During the Epic BT measurements, first, the baseline with HBSS-HEPES buffer (30 μl) was recorded for approximately 40–60 min. Following the stabilization of the biosensor signal, the measurement was stopped and the buffer was replaced with 30 μl of the desired coating solution and incubated for 30 min while gently shaking at room temperature. The biosensor plate was then replaced into the RWG imager and the signal was recorded. The coating solutions were then removed and the wells were rinsed three times with 30 μl of HBSS-HEPES buffer. Wells were then given 30 μl HBSS-HEPES buffer for the fourth time to establish a new baseline^27^. Then the EGCg solutions were pipetted (30 μl) into the wells and we measured them for an hour. Then a washing period came for 4 times, and the kinetic curves were recorded for 30 min again with HBSS-HEPES (30 μl). During this time, HeLa cells were brought into suspension by using pre-warmed trypsin-EDTA solution. Trypsin-EDTA was removed before complete detachment of HeLa cells and its activity arrested by adding culture medium (containing 10% FBS). Harvested cells were centrifuged at 380 g for 6 min and the cell pellet was resuspended in assay buffer with intensive pipetting. Cells were then counted in a hemocytometer, and 12000 cells were added to each sensor well. Wells for buffer control were also coated with the copolymers and treated in the same way as the sample wells during the experiment, except that they received assay buffer instead of cell suspension. All measurements were done in triplicates using three different wells at room temperature. Cell spreading was monitored until saturation of the biosensor signals (2 h). Averaging every 5 subsequent data points, the effective sampling rate was 1/15 s^−1^ 27.

### Calculation of surface adsorbed mass density from the OWLS and EpicBT biosensor data

OWLS monitors the effective refractive indices of the zeroth order transverse electric and transverse magnetic waveguide modes. Using these values, the optogeometrical parameters of the adsorbed layer can be calculated in a straightforward manner^9^,^48^,^51^,^52^. These are the thickness and refractive index of the added layer (*n*_*A*_, *d*_*A*_). After, the adsorbed mass surface density can be calculated by the well known Feijter’s formula:


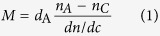


*n*_*C*_ is the refractive index of the medium covering the added layer and *dn/dc* is the refractive index increment of the solution from where the biomolecules adsorb. A value of 0.182 cm^3^/g can be used for most of the protein adsorption calculations^9^. It should be noted that the refractive index increment of the EGCg solutions deviated from this value, and therefore the following correction equation was applied.


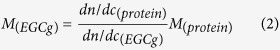


Here, the *dn/dc*_(protein)_ is 0.182 cm^3^/g and the *dn/dc*_(EGCg)_ is 0.21 cm^3^/g, measured by using a Rudolph refractometer.

The surface adsorbed mass can be also calculated from the raw Epic BT data. In this case the wavelength shift (Δλ in pm) can be transformed to surface adsorbed mass density (in ng/cm^2^) by using the following calibration equation established earlier by us^50^.


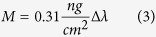


Note, this equation is valid for materials with *dn/dc*=0.182 cm^3^/g. The *dn/dc* value of the EGCg and oxidized EGCg solution was measured to be 0.21 cm^3^/g, so the above value needs to be divided by 1.154.

### Semiempirical quantumchemical method

Comparative modelling study on binding of PEG-fragment to EGCg and its oxidized dimer form was performed using the PM6 algorhytm as implemented in Gaussian 09 program package^53^,^54^.

### Long term effects of the EGCg exposed coatings studied by MTT (tetrazolium) assay

Coating procedure was performed in a 96-well suspension plate (831835500. Sarstedt AG & Co., Nümbrecht, Germany)with PP and PP:PPR as described above. The prepared coatings were treated by EGCg and oxidized EGCg solutions (concentrations: 1.953–2000 μg/ml, solved in HBSS-HEPES buffer) for 70 minutes. Then the EGCg and oxidized EGCg solutions were removed and the wells were rinsed three times with 50 μl of HBSS-HEPES buffer. Control cells were treated with HBSS-HEPES buffer. HeLa cells were plated into the coated and treated wells with initial cell number of 5000 per well and the cells were incubated in DMEM medium containing 10% fetal calf serum (FCS), 2 mM L-glutamine, 160 g/ml gentamycin at 37 °C. After 72–80 h incubation the cells were washed three times with serum free medium and the cytostatic effect of EGCg and oxidized EGCg was determined by 3-(4,5-dimethylthiazol-2-yl)-2,5-diphenyltetrazolium bromide (MTT)-assay^55^,^56^. 45 μl MTT-solution (2 mg/ml) was added to each well. The respiratory chain and other electron transport systems reduce MTT and thereby form non-water-soluble violet formazan crystals within the cell^57^. The amount of these crystals can be determined spectrophotometrically^58^. After 3 hrs of incubation cells were centrifuged for 5 min (380 g) and supernatant was removed. The obtained formazan crystals were dissolved in DMSO and optical density (OD) of the samples was measured at 540 and 620 nm using ELISA Reader (iEMS Reader, Labsystems, Finland). OD_620_ values were substracted from OD_540_ values. The percent of cytostatic effect was calculated using the following equation:


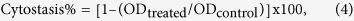


where OD_treated_ and OD_control_ correspond to the optical densities of the treated and the control cells, respectively. Two independent experiments were carried out with 3 parallel measurements. If reached, the 50% inhibitory concentration (IC_50_) values were determined from the dose-response curves. Usually the employed concentrations considered non-cytostatic when the 50% inhibitory level is not reached. The curves were defined using MicrocalTM Origin1 (version 8.6) software.

## Results and Discussion

### EGCg adsorption on the synthetic polymer coatings

First, the EGCg adsorption to the PP coating was investigated by using OWLS. Previous studies showed that PP has repellent properties. For instance, Lee and Spencer mentioned in their publication that a PP layer on various metal oxide surfaces displays resistance to non-specific adsorption of proteins and provides a lubricious surface in an aqueous environment^24^, furthermore, a pure PP coating completely blocks cell spreading: all cells remain small and round shaped, observed by Orgovan *et al*.^27^. However, the degree of nanoscale order of RGD motifs on a surface (PPR) has been shown to have a serious impact on cell spreading^27^. RGD is found within the ECM and has the ability to bind to cells via specific cell-surface receptors. Earlier studies showed that PPR reduced non-specific protein adsorption while promoting cell (fibroblasts) adhesion^59^. Later, Tosatti and coworkers observed that osteoblast cell number was restored on PP surface having RGD motif^60^. Increased cell spreading was observed on the PPR surfaces with the higher RGD density and the authors argued for reduced differentiation of osteoblasts to an extent conducive to proliferation rather than stimulating differentiation.

More recently, the cell behavior has been examined on PPR surface as a function of its nanotopography^61^ or the RGD surface density^27^. The PP and its cell adhesive, functionalized counterpart, PPR solutions can be mixed to increase the ligand density and to increase cell spreading^27^.

**Figure 1:**
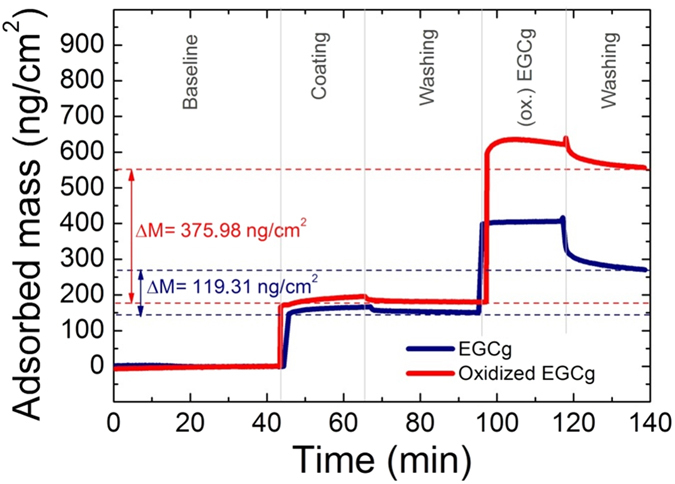
The *in situ* OWLS measurement on the adsorption of the PP coating, and the adsorbed mass curves of 500 μg/ml EGCg and oxidized EGCg on these coatings. The amounts of irreversibly bound EGCg and oxidized EGCg are indicated.

Despite the above data, we found that the PP coating strongly interacted with EGCg. Using *in situ* OWLS measurements (see Fig. 1), we proved that EGCg molecules bind irreversibly to the PP coating. The irreversibly bound material is more than 100 ng/cm^2^, greatly exceeding the monolayer surface coverage of EGCg molecules (around 30 ng/cm^2^). Moreover, the bound amount of the oxidized EGCg was more than three times of what was obtained for the non-oxidized, freshly prepared EGCg solution.

**Figure 2:**
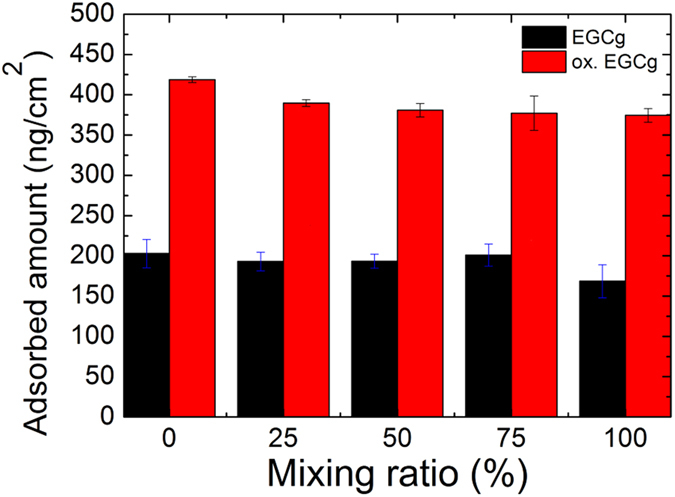
Adsorbed amount of EGCg and oxidized EGCg (500 μg/ml) on the copolymer coatings with varying RGD surface densities. 0% corresponds to the PLL-g-PEG and 100% corresponds to the purely PLL-g-PEG-RGD coating. The exact RGD surface densities for the various mixing rations are found in the publication of Orgovan *et al*.^27^.

We repeated these measurements using the PP:PPR coating as well, and got almost the same adsorbed values using OWLS (data not shown). To better illuminate the role of the RGD motifs, a more systematic experiment was run using the Epic BT biosensor. We performed experiments with coatings containing different RGD motif ratios to investigate, whether the density of the RGD motif affect the adsorption of EGCg molecules or not. Of note, a bit higher adsorbed amount of EGCg and oxidized EGCg could be observed in the case of PP coating (0% RGD), and a moderate decreasing tendency towards the higher RGD motif ratio can be demonstrated as well (see Fig. 2). However, compared to the total adsorbed amount, there are no significant differences in the adsorbed amounts of EGCg or oxidized EGCg (500 μg/ml) onto the coatings with various RGD motif densities. Semiempirical quantumchemical method also revealed, that the hydrogen bonding of EGCg and oxidized EGCg to PEG chain is much more significant in our system than to the region with RGD motif (see details later).

Although these studies demonstrate the strong interaction of EGCg with the copolymer films, for more detailed studies aiming at the concentration dependence of these interactions and the adhesion of living cells on these films, we employed the high-throughput Epic BT device.

### Concentration dependence of polyphenol binding and cell repellent properties of the copolymer coatings after EGCg exposure - Epic BT measurements

**Figure 3:**
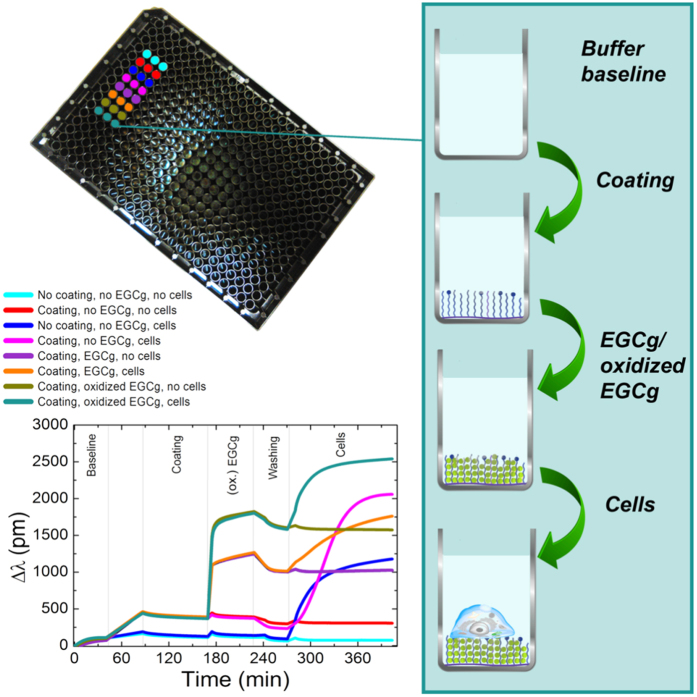
*In situ* kinetic curves recorded by the Epic BT instrument. A 384-well plate used in the experiment is also shown, together with the manipulation steps in a typical well (right scheme). Typical experimental curves are plotted (bottom left corner) for PP:PPR coating and 500 μg/ml EGCg concentration. The detailed experimental conditions corresponding to the various kinetic curves are indicated above the graph.

In order to in-depth investigate the above findings, the polymer coating process, the treatments with polyphenols and the subsequent cell adhesion on the coatings were on-line monitored by the high-throughput Epic BT biosensor, using various polyphenol concentrations and triplicates. Typical measurement curves in case of the PP:PPR coating are shown in Fig. 3. Here, only the curves with or without the 500 μg/ml EGCg or oxidized EGCg exposure are shown. One can see that the copolymer mixture irreversibly adsorbed on the biosensor surfaces, creating a cell adhesive coating (see the sigmoidal spreading curve when the coating was not exposed to polyphenol). The biosensor measurements verified that the EGCg molecules adsorbed on the copolymer coatings, and a significant amount remained on the films after the washing procedure. It is striking to see that the EGCg exposure changed the kinetics of cell adhesion recorded on the polyphenol exposed films. We measured the adhesion processes of the cells for two hours, because during this period these types of cells can reach a fully spread morphology on an adhesive surface^27^. Typical curves obtained after cell addition are also plotted in Fig. 3. Note, for active cell adhesion and spreading a sigmoid-like kinetic curve is observed (PP:PPR surface), while the non-specific cell adhesion results in an adsorption-like kinetic curve (bare surface) (see Fig. 3)^27^. Next, the PP and PPR coatings and the effects of polyphenol exposure on the cell repellent and cell adhesive properties of these films were investigated separately.

### PP coating exposed to EGCg and subsequent cellular adhesion

**Figure 4:**
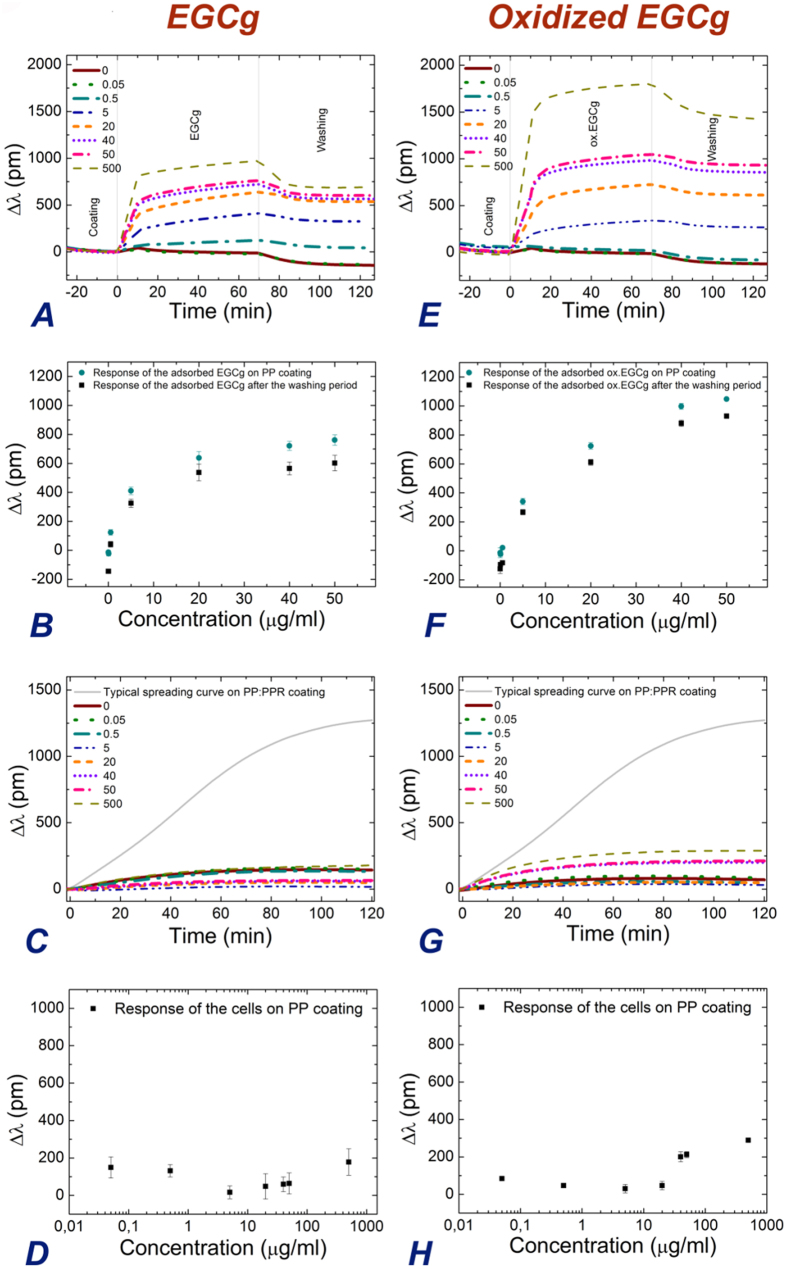
The recorded kinetic curves when the PP coating was exposed to EGCg and oxidized EGCg solutions with various concentrations (**A,E**). The concentration dependence of the finally adsorbed amounts are shown separately (**B,F**). The biosensor signals were recorded after cell addition onto the polyphenol exposed coatings. For comparison, the cell spreading curves recorded on the PP:PPR cell adhesive coating are also shown (**C,G**). The applied concentrations of EGCg and oxidized EGCg were 0, 0.05, 0.5, 5, 20, 40, 50, 500 μg/ml and indicated in the graphs. The concentration dependence of the cell adhesion signals after 2 hours of cell addition (**D,H**).

First, the PP coating was investigated in detail. The corresponding experimental results are shown in Fig. 4. For simplicity, the polymer coating process is not shown. It is seen that both the EGCg and oxidized EGCg molecules adsorbed on the PP coating, and a significant amount bound irreversibly and could not be removed by the washing procedure (see Fig. 4A,B,E,F). The effect was more pronounced in case of the oxidized EGCg (see Fig. 4A,E). Increasing the polyphenol concentration the bound amount was also increasing, but saturated after at around 20 μg/ml concentration in case of the EGCg (see Fig. 4B), and at around 40–50 μg/ml in the case of oxidized EGCg (see Fig. 4F). No significant cell adhesion signal was observed, except for the oxidized EGCg with the highest concentration, but even in this case the effect was not significant (Fig. 4C,G,D,H). Therefore, these experiments prove that the PP coating remains cell repellent, even after polyphenol exposure.

### PP:PPR coating exposed to EGCg and subsequent cellular adhesion

**Figure 5:**
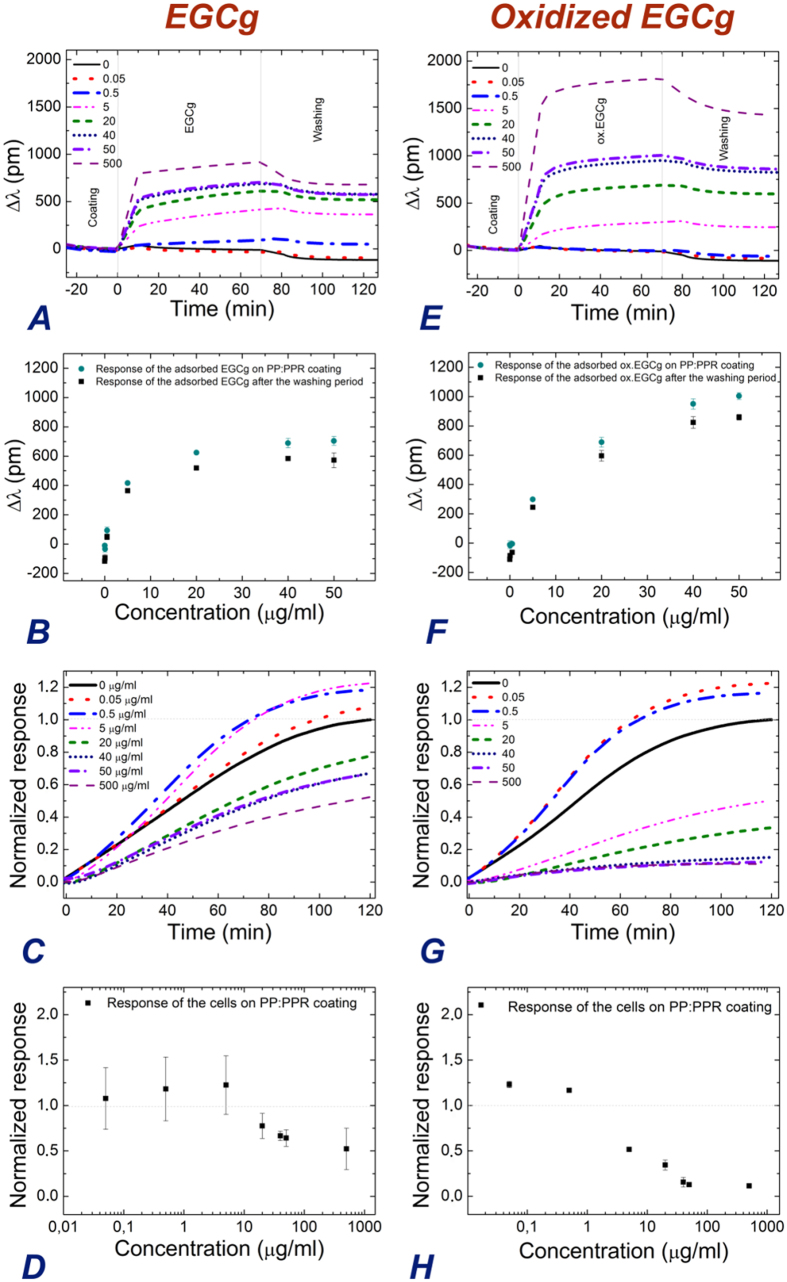
The recorded kinetic curves when the PP:PPR coating was exposed to EGCg and oxidized EGCg solutions with various concentrations (**A,E**). The concentration dependence of the finally adsorbed amounts are shown separately (**B,F**). The biosensor signals recorded after cell addition onto the polyphenol exposed coatings. For comparison, the cell spreading curves recorded on the bare PP:PPR cell adhesive coating are also shown and all of the cell adhesion curves are normalized (**C,G**). The applied concentrations of EGCg and oxidized EGCg were 0, 0.05, 0.5, 5, 20, 40, 50, 500 μg/ml and indicated in the graphs. The concentration dependence of the normalized cell adhesion signals after 2 hours of cell addition (**D,H**).

Next, the RGD displaying coating was investigated in a systematic manner. Similarly to the above results obtained with the PP coating, the EGCg and oxidized EGCg molecules irreversibly adsorbed on the PP:PPR coating. The RGD motif had a negligible influence on the polyphenol adsorption (see Fig. 5A,B,E,F). In contrast, the cell adhesiveness of the RGD displaying coating was significantly influenced by the polyphenols. As expected, without the polyphenol exposure a sigmoidal spreading curve was recorded^27^. But, in contrast to the PP coating, the magnitude of the cell adhesion and spreading was significantly affected by the polyphenol exposure (see Fig. 5C,G). Interestingly, for the lowest concentrations of EGCg and oxidized EGCg the cell adhesion was even slightly increased due to the polyphenol (see Fig. 5H,D). For higher concentrations, a clear decrease in cell adhesion was observed, and in case of oxidized EGCg, the adhesion and spreading on the polyphenol exposed coatings were completely absent. This behavior is further emphasized in Fig. 5D and H, where the effect is shown in the function of polyphenol concentration.

### The amount of polyphenol bound to the copolymer coatings

In order to understand the above observations, the bound amounts of polyphenol on the copolymer coatings were calculated from the biosensor data. As a result, using the OWLS data, on the PP coating the bound EGCg was determined to be 119.31 ng/cm^2^, while the bound mass per area of the oxidized form was 375.98 ng/cm^2^ (see also Fig. 1). Approximately the same values were obtained on PP:PPR coating (data not shown). Since these values greatly exceeds the estimated monolayer surface coverage for EGCg, the polyphenols form multilayers either on top or inside the copolymer coatings. Similar conclusions were reached using the Epic BT data, and these data were used to investigate the concentration dependence of the multilayer formation.

First, the number of formed EGCg and oxidized EGCg layers can be easily calculated by taking the geometrical parameters of an EGCg molecule (approx. 1.4 nm in size) and its molecular weight (458.37 g/mol). The results of these straightforward calculations are summarized in Table 1 and 2 for the PP and PP:PPR coatings, respectively, for three representative concentrations.

**Figure 6:**
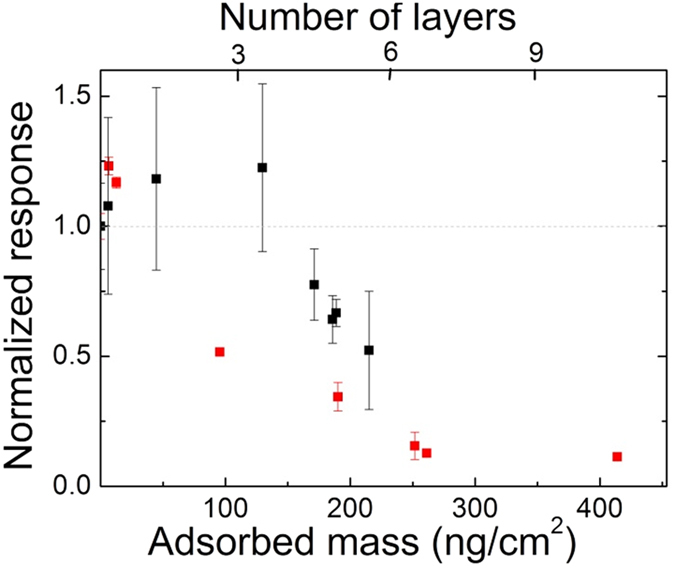
The normalized cell adhesion signal at saturation measured on the polyphenol exposed coatings as a function of bound polyphenol amount (bottom axis) and the calculated bound polyphenol layer number (top axis). Black squares: EGCg, red squares: oxidized EGCg.

From these data, it can be clearly seen, that the adsorbed polyphenol mass for the two types of coatings were almost the same, the RGD motif had practically no influence on layer formation. Depending on the concentration, multilayers formed and the number of layers could reach even 11 for the highest concentration of the oxidized EGCg. It is important to note, the maximum number of layers for EGCg was roughly half of this (5–6 layers). In order to better visualize the above results, the dependence of cell adhesion and spreading on the bound polyphenol amount and calculated layer numbers are plotted in Fig. 6.

While the multilayer formation is well evidenced, it is not clear whether multilayers are formed on top of the copolymer coatings or the polyphenols can diffuse and form multilayers inside the copolymer films. Importantly, looking at the measured cell adhesion data (see Fig. 5), we can safely exclude the possibility that compact polyphenol layers are formed on the top of the copolymer films. For example, taking the 5 μg/ml concentration, when two layers are formed (see Table 1 and 2), the HeLa cells still can adhere and spread on the films. This seems to contradict to compact multilayer formation only at the top of the films, since two polyphenol layers could completely block the RGD motifs. This suggests that the polyphenols start to adsorb inside the polymer coating. Moreover, this hypothesis is supported by the fact that the PEG side-chains are heavily hydrated. The water content of the PEG chains are 80 ± 6 wt%^25^. By supposing that all of the bound water is exchanged to polyphenol, one can estimate the maximum possible polyphenol amount bound inside the films. Taking the OWLS data from Fig. 1, this estimated maximum amount is around 560 ng/cm^2^. Therefore, the measured maximum bound polyphenol amounts (see Table 1 and 2) would correspond to a packaging density of around 35–70%, which is highly realistic.

### Modeling of EGCg binding to PEG fragment - the H-bonding ability of the compounds

One could reasonably expect, that the roughly two times maximally adsorbed amount of oxidized EGCg compared to the fresh EGCg is in close connection with the dimerization of the molecules, and that EGCg and its oxidative products^45^ bind to the PEG chains by hydrogen bonds. Calculations based on a semiempirical quantumchemical method well supported our hypothesis.

**Figure 7:**
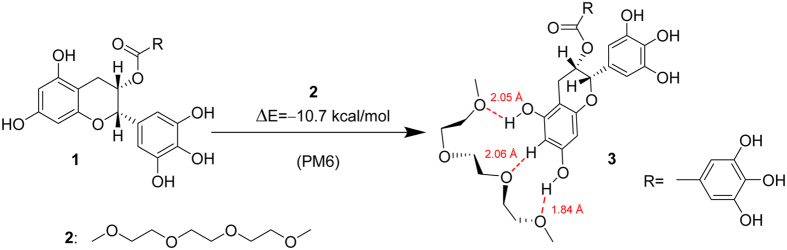
Modelling study on the complex formation between EGCg (**1**) and 2,5,8,11-tetraoxadodekane (**2**) representing a possible bonding domain of PEG. Geometry optimization and energy calculation were carried out by PM6 semiempirical quantumchemical method.

**Figure 8:**
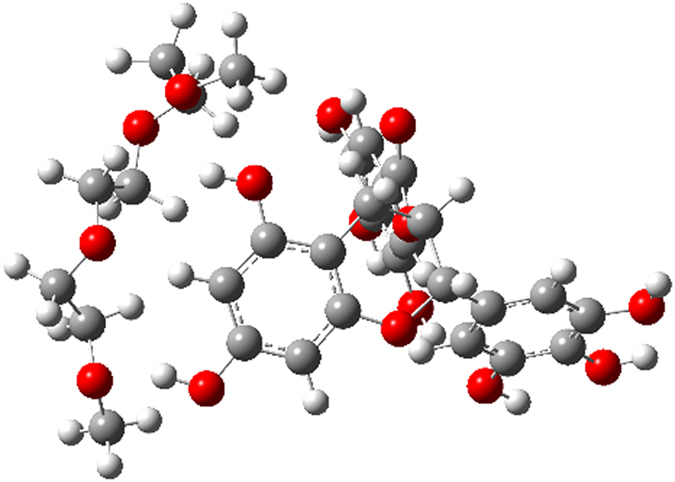
Optimized structure of complex 3 stabilized by H-bonds.

**Figure 9:**
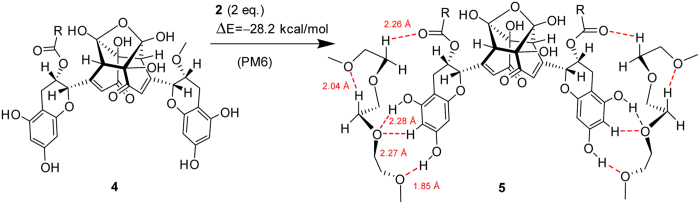
Modelling study on the complex formation between the assumed oxidized EGCg dimer (4) and two equivalents of 2,5,8,11-tetraoxadodekane (2) carried out by PM6 semiempirical quantumchemical method.

**Figure 10:**
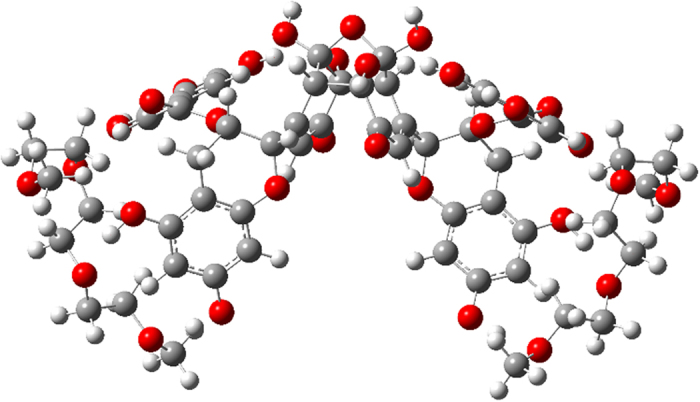
Optimized structure of complex **5** stabilized by a reorganized system of H-bonds.

The calculated energetics unambiguously indicated that, in agreement with the experimental findings, that dimer **4** resulted from the oxidation of EGCg features a significantly stronger H-bonding-based binding to a PEG fragment than does the intact EGCg (see details in Figs 7, 8, 9, 10). It must be pointed out that 14.1 kcal/mol (ΔE = 28.2 kcal/mol) can be assigned as a change in the total energy to the binding of a single molecule of **2.** The difference between the calculated energetics (ΔE = 14.1 kcal/mol – 10.7 kcal/mol = 3.4 kcal/mol) can be regarded as the measure of the increased PEG-binding propensity of oxidized dimer EGCg relative to that of EGCg.

Due to the formation of hydrogen bonds the calculated change in the energetics refers to a favorable exothermic character of the complex formation.

**Figure 11:**
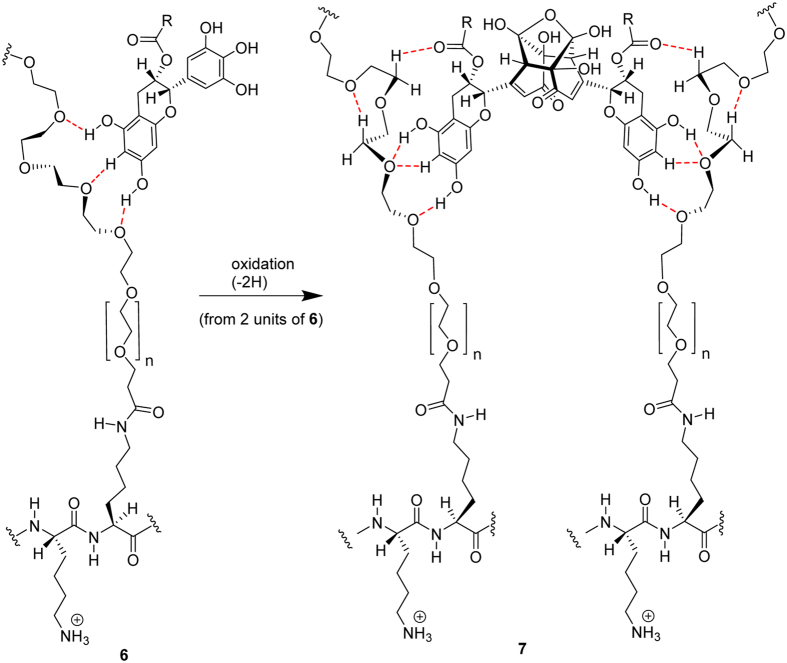
A series of oxidative dimerization might give rise to an increase in the degree of organization of the EGCg-bonded PEG chains in the surface attached copolymer films. This cross-coupling of polymer chains makes the layer more compact and rigid.

Moreover, it has to be emphasized that a series of oxidative dimerization might give rise to an increase in the degree of organization of the EGCg-bonded PEG chains, making the PLL-*g*-PEG system more compact with the assembly of well-defined hydrogen bonding network of enhanced stability (see details in Fig. 11). Such an interaction definitely makes the layer more dense and rigid.

### The proposed mechanism and long term control experiments

**Figure 12:**
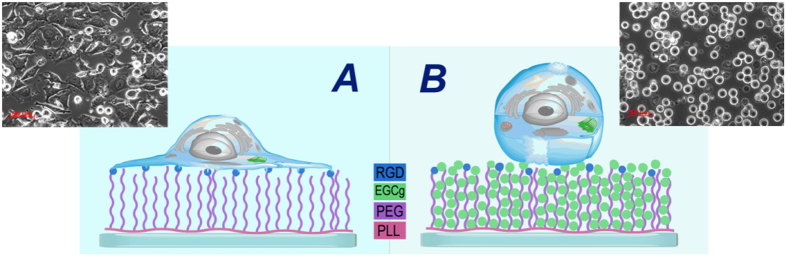
Schematic illustration of the proposed mechanisms on polyphenol binding and HeLa cell adhesion on the copolymer films. (**A**) The cell can easily spread on the PP:PPR coating because the RGD motifs are accessible. (**B**) EGCg molecules bind to the PP:PPR chains, block the RGD motifs and inhibit cell adhesion. The observed morphologies of the cells can be also seen in the images taken by phase contrast microscopy.

An estimation on the capacity of the coatings can be also made by taking the reported chain lengths of the PEG molecules. The calculated number of layers coincides well with a model supposing that EGCg binds inside the PEG chains by hydrogen bonding. The compressible thickness of the PEG polymer layer (7 ± 0.5 nm^25^) and the size of an EGCg molecule (1.4 nm) suggest that 5–6 molecules of EGCg can bind to a PEG chain. The spacing between the PEG chains is 1–2 nm^25^, so the EGCg molecules fit well into the spacing of the coating. Note, adhesion decreases drastically when the number of layers are 5–6 (see Fig. 5), presumably reaching the RGD motifs. The suggested binding mechanisms, with its effects on cell adhesion, are schematically summarized in Fig. 12. Taking all together, the polyphenol molecules adsorb between the PEG chains by H-bonds and form multilayers. For the highest concentrations these layers can even reach the top of the copolymer films, where effectively block the RGD motifs, decreasing cell adhesion and spreading on these functional coatings (see Figs 4 and 5).

It is interesting to revisit the finding, that the low concentrations of EGCg (0.05, 0.5, 5 μg/ml) and oxidized EGCg (0.05, 0.5 μg/ml) caused greater cell adhesion compared to the cell adhesion without polyphenol pretreatment (see Figs 4 and 5). The reason can be multifarious, but building on the proposed adsorption mechanism, we suppose that low concentrations of EGCg and oxidized EGCg decrease the water content and increase the rigidity of the coatings. The access to the RGD motif is not influenced, because we suggest that the molecules first bind in between the PEG chains, to the region near the substrate. This hypothesis is in full agreement with the study by Schlunck *et al*., where the authors found that ECM rigidity modulated the cell spreading and focal adhesion size. Furthermore, serum-induced extracellular signal-regulated kinase (ERK) phosphorylation and focal adhesion kinase (FAK) activation increased with rising substrate rigidity^62^. This is an important finding, since the rigidity of matrix substrates determines not just cell adhesion, but growth, differentiation and motility, too^63^,^64^,^65^,^66^,^67^,^68^.

**Figure 13:**
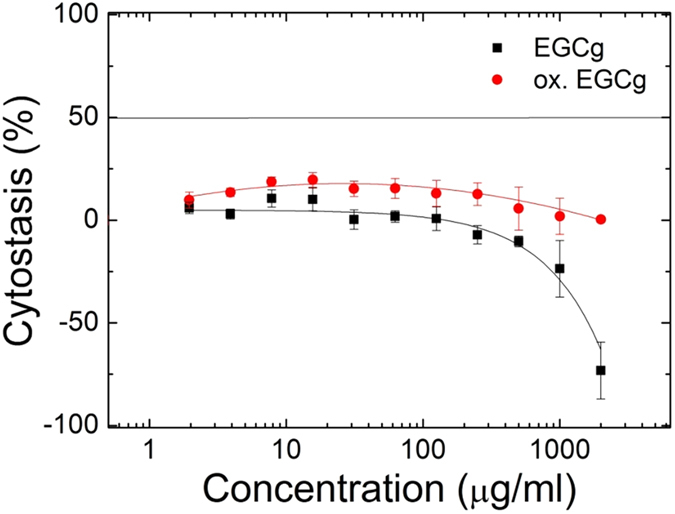
Cytostatic effects of EGCg and oxidized EGCg treated PP:PPR coating after 72–80 h after the treatment. The treated coating had no cytostatic effect on the HeLa cells, but the proliferation rate was faster with increasing EGCg concentration.

In order to better illuminate the above results, we have run long-term cytostatic experiment using EGCg and oxidized EGCg pre-exposed substrates. The results are shown in Fig. 13. The negative cytostatic values show that the cells more intensively proliferate on the polyphenol pre-treated coatings. Therefore, we found that in the long term (several days) the adsorbed polyphenol facilitated the proliferation of the cells.

This is in line with Epic BT adsorption data where we measured the oxidized EGCg (500 μg/ml) capacity of the layers and found that after one day of washing in EGCg free buffer roughly 2 layers of EGCg was removed from the previously EGCg saturated coatings. Therefore, in long-term the RGD motifs became accessible for the cells, but the non-removed, irreversibly bound EGCg contributes to the rigidity of the layer and facilitates proliferation. It should be noted that during this long term experiment the polymer adsorbed EGCg might gets oxidized as well; and the adsorbed polyphenols probably also facilitate the interaction with materials secreted by the cells.

According to our proposed model, the weaker binding of oxidized EGCg experienced at the upper layers (where the RGD motifs are present) can be accounted for the enhanced conformational mobility (flexibility) of the distant segments of the pending PEG chains, which must be highly restricted by the cross-coupling complexation accompanied by a substantial decrease in the overall entropy of the whole system. On the other hand, a much smaller change in entropy is obviously expected to slightly influence the complexation of the more rigid PEG-regions positioned at the lower layers of the investigated macromolecular assembly.

## Conclusions

In the present work, we highlighted the possibilities offered by high-throughput label-free biosensors in cell adhesion research. We fabricated layers from PLL-*g*-PEG and PLL-*g*-PEG-RGD polymers, and then observed the binding of EGCg inside these coatings. After that, cell addition to the EGCg treated coatings was also performed. An important novelty of this study is that the polymer coating fabrication, its treating with small molecule, and the observation of cell adhesion could be all studied on-line by the Epic BT label-free biosensor in triplicates, and with different EGCg and oxidized EGCg concentrations. With this novel, multicomponent model system, we got representative kinetic curves of the processes. The application of the proposed methodology opens up new avenues in cell adhesion research. Doing the same amount of experiments by a biosensor, with a single sensor (like OWLS), would have been taken for weeks or maybe months. Conversely, this time is only few hours by employing the high-throughput Epic BT. Also, the present sensor is sensitive to sub-nanometer scale changes in cell membrane positioning or protein distribution, while averaging the signals of thousands of adhering cells. This remarkable sensitivity and throughput is not in the reach of microscopy. Another advantage of the proposed method is that both the small molecules and the much larger cells could be easily investigated in the same experiment with very high resolution. This combination is usually not feasible with other type of methods.

As an important result, we observed that both the EGCg and oxidized EGCg molecules adsorbed on the PLL-*g*-PEG coating, and a significant amount bound irreversibly, could not be removed by the washing procedure with EGCg free buffer. The effect was more pronounced in case of the oxidized EGCg. Increasing the polyphenol concentration the bound amount was also increasing, but saturated after at around 20 μg/ml concentration for EGCg, and at around 40–50 μg/ml for oxidized EGCg. No significant cell adhesion signal was observed on the treated films, except for the oxidized EGCg with the highest concentration, but even in this case the effect was rather small. Therefore, these experiments prove that the PLL-*g*-PEG coating remains cell repellent, even after polyphenol exposure. Similarly to the above results obtained with the PLL-*g*-PEG coating, the EGCg and oxidized EGCg molecules irreversibly adsorbed on the PLL-*g*-PEG:PLL-*g*-PEG-RGD coating. The RGD motif had a negligible influence on the polyphenol adsorption. In contrast, we found that the cell adhesiveness of the RGD displaying coating was significantly influenced by the polyphenols. As expected, without the polyphenol exposure a sigmoidal spreading curve was recorded. However, in contrast to the PLL-*g*-PEG coating, the magnitude of the cell adhesion and spreading was significantly affected by the polyphenol exposure. Importantly, for the lowest concentrations of EGCg and oxidized EGCg, the cell adhesion was even slightly increased due to the polyphenol pre-treatment of the films. For higher concentrations, a clear decrease in cell adhesion was observed, and in case of the oxidized EGCg the adhesion and spreading on the polyphenol exposed coatings were completely absent. The bound amounts of polyphenol on the copolymer coatings were calculated from the OWLS and Epic BT data. Based on the calculated values, we suggested multilayer formation onto both copolymers. The number of EGCg and oxidized EGCg layers were calculated using the geometrical parameters of the EGCg molecule and its molecular weight. As a result, in the case of 500 μg/ml EGCg, approximately 5 layers, while in the case of 500 μg/ml oxidized EGCg, 10-11 layers could be calculated on both coatings. This data suggested that the dominant oxidative product during the polymer-polyphenol interactions is presumably the EGCg dimers. By combining these findings with the measured cell adhesion data, we proposed that the polyphenol molecules bound in between the PEG chains of the copolymer coatings and form multilayers. At high concentrations the formed multilayers can effectively block the RGD motifs, decreasing cell adhesion and spreading on the polyphenol exposed films. We also found, that low concentrations of the polyphenols slightly increase the cell adhesiveness of the coatings, which we attributed to a decreased water content and increased polymer rigidity due to polyphenol binding. Data of long-term cytostatic studies fitted to these results. Using a semiempirical quantumchemical method, we also showed that EGCg binds to the PEG chains of PLL-*g*-PEG and PLL-*g*-PEG-RGD by hydrogen bonds. The calculations illuminated the differences in binding affinity between the fresh and oxidized EGCg and well supported the experimental finding that the binding is stronger for the oxidative products of EGCg. In case of the dimer oxidative product of EGCg we proposed a cross-coupling mechanism of polymer chains by a hydrogen-bonding network.

Besides emphasizing the novel potentials of high-throughput label-free biosensors in cell adhesion science, our work opens up new directions in polyphenol research, too. The effects of EGCg oxidization is often neglected in present literature^46^, but were systematically investigated here. Moreover, the vast majority of EGCg literature deals with the direct influence of EGCg on the living cells, but the indirect influence of polyphenols, through changing the adhesion matrix of the cells, is very rarely investigated^29^,^69^,^70^. The present systematic study highlighted the importance of this possibility. We showed that EGCg can block important cell adhesion ligands and directly affect matrix properties by hydrogen bonding. The introduced methodology could be extended to extracellular matrix components and to other small molecules in a straightforward manner. We believe that the employed PLL-PEG-RGD system is simple, but illuminates the most important features of EGCg-adhesion matrix interactions. Therefore, the introduced model of EGCg action on cell adhesion ligand accessibility, matrix rigidity, and cross-coupling of various segments by EGCg through hydrogen bonds might be relevant for other, more complicated *in vivo* systems, too.

## Additional Information

**How to cite this article:** Peter, B. *et al*. Green tea polyphenol tailors cell adhesivity of RGD displaying surfaces: multicomponent models monitored optically. *Sci. Rep.* **7**, 42220; doi: 10.1038/srep42220 (2017).

**Publisher's note:** Springer Nature remains neutral with regard to jurisdictional claims in published maps and institutional affiliations.

## Figures and Tables

**Table 1:** The raw biosensor data, the calculated adsorbed polyphenol mass and the number of formed polyphenol layers on the PP coating for three different concentrations.

	Conc. (μg/ml)	Δλ (pm)	ΔM (ng/cm^2^)	Number of layers
**EGCg**	5	469.55 ± 48.97	126.57 ± 13.20	3.34 ± 0.34
	50	747.13 ± 92.20	201.4 ± 24.85	5.33 ± 0.65
	500	834.71 ± 108.61	225 ± 29.26	5.95 ± 0.77
**Ox. EGCg**	5	391.31 ± 31.75	105.48 ± 8.55	2.79 ± 0.22
	50	1053.95 ± 28.21	284.1 ± 7.60	7.51 ± 0.20
	500	1543.09 ± 17.47	415.96 ± 4.7	11 ± 0.12

**Table 2:** The raw biosensor data, the calculated adsorbed polyphenol mass and the number of formed polyphenol layers on the PP:PPR coating for three different concentrations.

	Conc. (μg/ml)	Δλ (pm)	ΔM (ng/cm^2^)	Number of layers
**EGCg**	5	480.94 ± 4.81	129.64 ± 1.29	3.43 ± 0.03
	50	689 ± 87.49	185.73 ± 23.58	4.91 ± 0.62
	500	797.39 ± 140.50	214.94 ± 37.86	5.68 ± 1.00
**Ox. EGCg**	5	354.3 ± 8.24	95.5 ± 2.21	2.52 ± 0.05
	50	969.04 ± 33.55	261.21 ± 9.04	6.91 ± 0.23
	500	1535.54 ± 41.46	413.92 ± 11.17	10.95 ± 0.29
